# 2771. Antibiotic Prescription Practices and Perceptions Among Physicians in Bangladesh

**DOI:** 10.1093/ofid/ofad500.2382

**Published:** 2023-11-27

**Authors:** Md Golam Dostogir Harun, Shariful Amin Sumon, Md Saiful Islam

**Affiliations:** icddrb, Dhaka, Dhaka, Bangladesh; icddr,b, Mohakhali, Dhaka, Bangladesh; University of New South Wales, Sydney, South Australia, Australia

## Abstract

**Background:**

Antimicrobial resistance (AMR) is a global public health threat, especially in low-resource settings. Irrational and inappropriate use of antibiotics in healthcare settings has led to widespread drug resistance. Antibiotics are widely and inappropriately used without adherence to international guidelines in Bangladesh, which may contribute significantly to the development of antimicrobial resistance (AMR). With the rapid raising of AMR, it is important to assess antibiotic prescribing perceptions and practices among physicians in Bangladesh.

**Methods:**

From September to December 2020, a hospital-based cross-sectional survey was conducted among 517 physicians in 11 tertiary care hospitals across Bangladesh. Face-to-face interviews were done to collect information. Descriptive statistics were performed to analyze the data and for interpretation

**Results:**

Nearly two-thirds (66 %) of the physicians considered AMR a significant concern. Over three-fourths (77%) opined that easy access to antibiotics without a prescription was the most important contributor to AMR, followed by overuse/inappropriate use (68%); whereas 42% of physicians reported inadequate diagnostic tests and one-fifth blamed poor infection control measures as the cause of rising AMR. The majority (77 %) of the participants reported following senior physicians’ guidance to prescribe antibiotics while 41.6% prescribe considering the affordability of patients _and_ antibiotic cost. All (100%) physicians stated that updated antibiotic use guidelines are necessary for the appropriate and rational use of antibiotics

**Conclusion:**

The assessment's findings can aid policymakers in the development and implementation of a national guideline for ensuring the rational use of antibiotics and enforcement of drug regulations at all stages, including the dispense of antibiotics in Bangladesh.

**Disclosures:**

**All Authors**: No reported disclosures

